# Does adding the drug–drug similarity to drug–target interaction prediction methods make a noticeable improvement in their efficiency?

**DOI:** 10.1186/s12859-022-04831-7

**Published:** 2022-07-14

**Authors:** Reza Hassanzadeh, Soheila Shabani-Mashcool

**Affiliations:** 1 Department of Engineering Sciences, Faculty of Advanced Technologies, University of Mohaghegh Ardabili, Namin, Iran; 2 Laboratory of Bioinformatics and Drug Design, Institute of Biochemistry and Biophysics, University of Tehran, Tehran, Iran

**Keywords:** Drug–target interaction, Drug repositioning, Machine learning, Drug–drug similarity

## Abstract

Predicting drug–target interactions (DTIs) has become an important bioinformatics issue because it is one of the critical and preliminary stages of drug repositioning. Therefore, scientists are trying to develop more accurate computational methods for predicting drug–target interactions. These methods are usually based on machine learning or recommender systems and use biological and chemical information to improve the accuracy of predictions. In the background of these methods, there is a hypothesis that drugs with similar chemical structures have similar targets. So, the similarity between drugs as chemical information is added to the computational methods to improve the prediction results. The question that arises here is whether this claim is actually true? If so, what method should be used to calculate drug–drug chemical structure similarities? Will we obtain the same improvement from any DTI prediction method we use? Here, we investigated the amount of improvement that can be achieved by adding the drug–drug chemical structure similarities to the problem. For this purpose, we considered different types of real chemical similarities, random drug–drug similarities, four gold standard datasets and four state-of-the-art methods. Our results show that the type and size of data, the method which is used to predict the interactions, and the algorithm used to calculate the chemical similarities between drugs are all important, and it cannot be easily stated that adding drug–drug similarities can significantly improve the results. Therefore, our results could suggest a checklist for scientists who want to improve their machine learning methods.

## Introduction

Most drugs fail in the early stages of a clinical trial and it takes a lot of time and cost for a drug to be successful in the market [1, 2]. These factors have led scientists to work on better and cheaper ways to find suitable drugs. One of the most effective and interesting solutions to solve these problems is drug repositioning (also called drug repurposing). It is true that drug repositioning, by eliminating the early stages of drug design, can speed up research, but it also has drawbacks. For example, determining the dosage of a drug that is considered for a new disease using drug repositioning is one of the most important challenges of this viewpoint because the drug has already been considered for another disease with a specific dose. However, this viewpoint has found its place and we have to consider it today.

One of the most important steps of drug repositioning is identifying Drug–Target Interactions (DTI), which is a difficult task if laboratory and traditional methods are used. In contrast, computational methods can be more effective both in terms of time and cost. These methods can identify or predict DTI more quickly. The computational methods are usually based on machine learning or recommender systems. To predict interactions, these methods first consider a mathematical model for the information in the databases and then add biological and/or chemical information to the model, according to the guilt by association principle. For example, NRLMF [3], NetLapRLS [4], BLM-NII [5], WNN-GIP [6] and DT-Hybrid [7] are some of the well-known methods in this field. In addition to the DTI problem, computational methods are also widely used to predict drug–drug interactions and drug–disease associations [8–10].

NRLMF is a matrix factorization approach that predicts the probability that a drug would interact with a target. In this method, the properties of a drug and a target are represented by two latent vectors in the shared low dimensional latent space, respectively [3]. NetLapRLS is a semi-supervised learning method based on Laplacian regularized least square. NetLapRLS, by incorporating a new kernel established from the known drug-protein interaction network, is actually an improvement of the LapRLS [4, 11]. The bipartite local model (BLM) is a supervised learning approach introduced by Bleakley and Yamanishi in 2009 [12]. To improve the BLM, Mei et al. presented a simple procedure called neighbor-based interaction-profile inferring (NII) and integrated it into the existing BLM method and called it BLM-NII [5]. WNN-GIP is actually a combination of a simple weighted nearest neighbor algorithm and the GIP method [6, 13]. An example of recommender systems method introduced for DTI prediction problem is DT-Hybrid. It is a network-based interface method that extends a well-established recommendation technique by domain-based knowledge including drug and target similarity [7]. Many other algorithms have been introduced for this problem, but the algorithms mentioned are the most popular and can be considered as the state-of-the-art methods in this field.

**Fig. 1:**
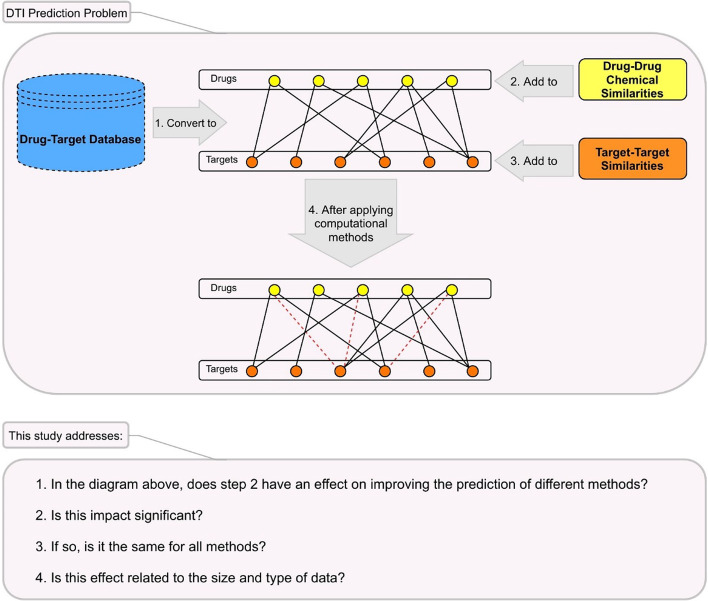
Schematic illustration of the DTI prediction problem and the questions addressed in this study

As mentioned, the methods first model the information in databases. There are some public databases, for example, KEGG [14], PubChem [15], DrugBank [16], and ChEMBL [17] that contain information about drugs, targets, and interactions between them. Usually, all methods introduced for predicting DTI interactions use DrugBank to evaluate their results or compare them to other methods. Regardless of what algorithm each of these methods uses, they all add similarity between targets and chemical structure similarity between drugs to improve the prediction (Fig. 1). The similarities between targets (proteins) are always calculated by the Smith-Waterman method [18] and the chemical structure similarities between drugs are usually computed with SIMCOMP [19] which has been implemented in the KEGG system for searching similar chemical structures in the chemical structure databases. SIMCOMP is a graph-based method and uses a graph alignment algorithm to get a global similarity score based on the size of the common substructures between two compounds [5]. Of course, other information such as molecular fingerprints can be used to calculate similarities between drugs, but it is not usually used. There are several types of molecular fingerprints (e.g., MACCS [20], PubChem fingerprint [21], BCI fingerprints [22] and TGD [23]). PubChem fingerprints are 2D fingerprints that make a drug to be expressed by a vector and used to discover similar conformers by the PubChem database. These fingerprints are very popular and easily calculated for every drug.

The purpose of this study is not to identify the best method. Here, we want to discuss the following questions specifically for the DTI prediction problem (Fig. 1):

- Does considering the similarity of drugs indeed improve the results of computational methods?
- Is SIMCOMP the best way to calculate drug–drug similarities in any computational method?
- Do the type and size of the dataset affect the improvement that occurs with adding drug–drug similarities?

In this paper, the similarity between drugs refers to the chemical structure similarity.

## Materials and methods

### Datasets

Yamanishi et al. have provided four benchmark drug–target interaction datasets including Nuclear Receptors, G-Protein Coupled Receptors (GPCR), Ion Channels, and Enzymes [24]. The datasets are publicly available at http://web.kuicr.kyoto-u.ac.jp/supp/yoshi/drugtarget/. The interactions were retrieved from databases KEGG BRITE [25], BRENDA [26], SuperTarget [27], and DrugBank [16]. These datasets of known DTIs are commonly considered as the gold standard for evaluating the performance of any new method introduced for DTI prediction problem. Each dataset contains three types of information in the form of matrices:

1. The drug–target interaction matrix, where the presence or absence of an interaction is indicated by 1 or 0, respectively.
2. The drug–drug similarity matrix calculated by SIMCOMP [19].
3. The target-target similarity matrix obtained by Smith-Waterman method [18].

To obtain the matrix mentioned in the second case, the chemical structure of a drug is treated as a 2D graph consisting of atoms as vertices and covalent bonds as edges. SIMCOMP provides the atom alignments between two chemical compound graphs, then it can also calculate the similarity of two chemical compounds by counting the number of matched atoms in those atom alignments. The calculation of similarity is based on the algorithm to solve the maximal common subgraphs of two graphs as the maximum vertex induced common subgraph or as the maximum edge induced common subgraph. The maximal common subgraphs of two graphs can be found by searching for maximal cliques in the association graph [19].

Some properties of datasets are shown in Table 1. The abbreviations in Table 1 are as follows:

- $N_{D}$: Number of drugs.
- $N_{T}$: Number of targets.
- $N_{I}$: Number of interactions.
- $$Density = {N_{I} }/{({N_{D} \times N_{T}) }}$$
- $AD_{T}$: Average number of drugs per target.
- $AT_{D}$: Average number of targets per drug.
- $D_{1T}$: Percentage of drugs with only one target.
- $T_{1D}$: Percentage of targets with only one drug.

**Table 1:** The properties of the benchmark datasets

Dataset	Nuclear Receptors	GPCR	Ion Channels	Enzymes
$N_{D}$	54	223	210	445
$N_{T}$	26	95	204	664
$N_{I}$	90	635	1476	2926
Density	0.0641	0.0299	0.0344	0.0099
$AD_{T}$	3.46	6.68	7.24	4.41
$AT_{D}$	1.67	2.85	7.03	6.58
$D_{1T}$	72.22%	47.53%	38.57%	39.78%
$T_{1D}$	30.77%	35.79%	11.27%	43.37%

### Evaluation

For each data set, in addition to the default drug similarity matrix obtained by SIMCOMP, we calculated 104 other matrices including one hundred random similarity matrices, one matrix where every element is equal to one, and three matrices calculated from PubChem 2D fingerprint using Tanimoto coefficient, Dice coefficient and Cosine similarity. The software PaDEL was used to obtain PubChem 2D fingerprints of all drugs [28]. For a drug, PubChem 2D fingerprints is a binary vector of length 881 that encodes the presence or absence of specific molecular substructures. Then, for the fingerprints of two drugs A and B, the Tanimoto, Dice and Cosine similarity can be calculated as follows:

- $$Tanimoto\left( {A,B} \right) = \frac{c}{a + b - c}$$,
- $$Cosine\left( {A,B} \right) = \frac{c}{{\sqrt {ab} }}$$,
- $$Dice\left( {A,b} \right) = \frac{2c}{{a + b}}$$
  , where $a$ equals the amount of bit set to 1 in A, $b$ equals the amount of bits set to 1 in B and $c$ equals the amount of bits set to 1 in both A and B.

We considered a matrix where every element is equal to one to find out what happens to the results of the algorithms if the similarity of the drugs is not affected. To show how far the effect of adding drug–drug similarity to DTI problem is from the random effect that may occur, we generated 100 random similarity matrices between drugs. To make the comparison fair, we consider four state-of-the-art methods NRLMF, NetLapRLS, BLM-NII and WNN-GIP. Therefore, in short, we executed every algorithm on every dataset using every drug–drug similarity matrix. To do this, we slightly modified the PyDTI package [3] to perform the evaluation. Like most studies in this field, results are assessed using the area under the ROC curve (AUC) and the area under the precision-recall curve (AUPR). Similar to [3, 4, 6, 13], we performed tenfold CV for five times to evaluate the performance of the methods on datasets. Then, we calculated the average AUC and AUPR over the five repetitions. In the next section, we will illustrate the results of the evaluations.

## Results and discussion

Before discussing the results, it is necessary to state some of the abbreviations given in the tables and figures as follows:

- All-onesSim: The value obtained for the matrix where every element is equal to one.
- MeanRandoms: The average value obtained for random matrices.
- BestRandom: The best value obtained for the random matrices.
- WorstRandom: The worst value obtained for the random matrices.
- CosinePF: The value obtained for the matrix calculated by Cosine similarity for the PubChem fingerprint.
- DicePF: The value obtained for the matrix calculated by Dice similarity for the PubChem fingerprint.
- TanimotoPF: The value obtained for the matrix calculated by Tanimoto similarity for the PubChem fingerprint.

The evaluation results on Enzyme, GPCR, Ion Channel and Nuclear Receptors datasets are shown in Tables 2, 3, 4 and 5, respectively. In these tables, higher value cells have a green color, middle value cells have a yellow color, and lower value cells have a red color. It is worth noting that the best parameters for each algorithm are obtained in [3], and we have used these parameters here as well.

**Table 2:**
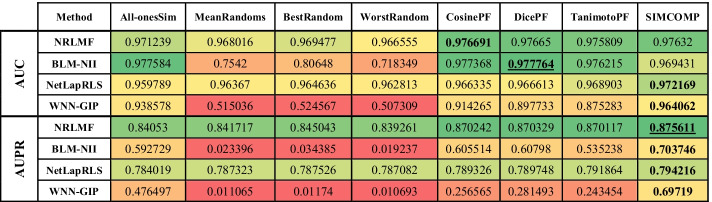
Comparing different drug–drug similarities on Enzyme dataset

**Table 3:**
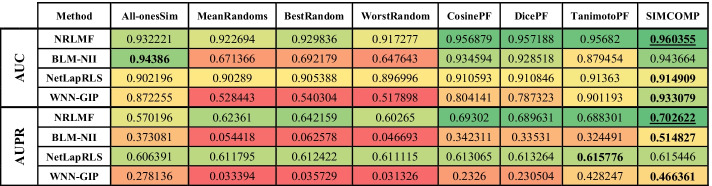
Comparing different drug–drug similarities on GPCR dataset

**Table 4:**
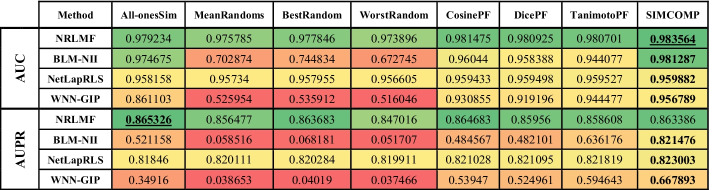
Comparing different drug–drug similarities on Ion Channels dataset

**Table 5:**
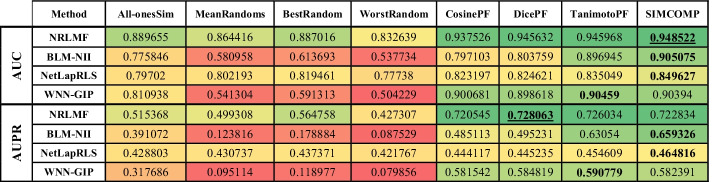
Comparing different drug–drug similarities on Nuclear Receptors dataset

In each row of tables, the best similarity matrix for each algorithm is bolded. The best AUC and AUPR are also marked with underlines. The first point about these tables is that the use of random matrices has degraded the efficiency of the methods. In fact, what the first four columns of the tables show is that ignoring the drug–drug similarities yields far better results than using inaccurate drug–drug similarities. It should be noted that the NRLMF and NetLapRLS have less tolerance than other methods in this case. Although the purpose of this study is not to identify a better method, the performance of NRLMF is better than other methods in most cases. In the Enzyme dataset (Table 2), the AUPR value for all methods and the AUC value for NetLapRLS and WNN-GIP methods are the best values when SIMCOMP similarity is considered. The NRLMF and BLM-NII methods obtain the best AUC value if they use the CosinePF and DicePF similarities, respectively. In the GPCR dataset (Table 3), the AUC for BLM-NII and the AUPR for NetLapRLS are the best values if they use the All-onesSim and TanimotoPF similarities, respectively. Except for these two cases, according to Table 3, the use of SIMCOMP has given the best results in all cases. Table 4 shows that, in the Ion Channels dataset, using All-onesSim for the NRLMF method leads to a better AUPR. In all other cases, it is clear that SIMCOMP is the best.

In the Nuclear Receptors dataset (Table 5), the SIMCOMP gives both the best AUC and AUPR for NetLapRLS and BLM-NII methods. The same thing happens with TanimotoPF and WNN-GIP. The AUC and AUPR values for NRLMF are the best if it uses the SIMCOMP and DicePF similarities, respectively. In summary, these tables show that in almost 94% of experiments, the use of drug–drug chemical structure similarities has led to better results.

**Fig. 2:**
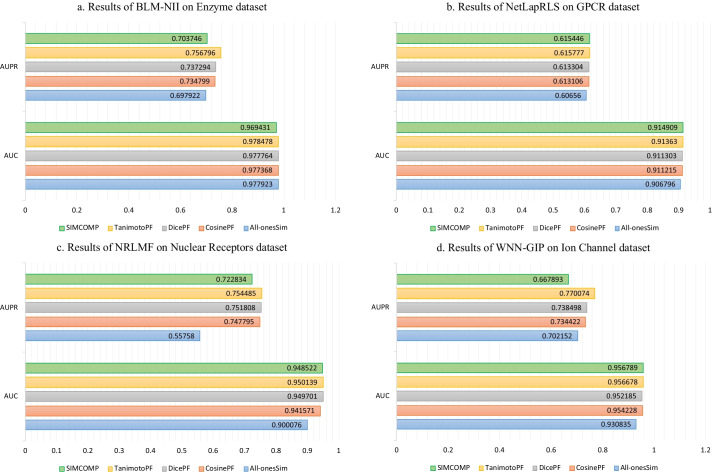
The AUC and AUPR values obtained by tuning the parameters of each method for different drug–drug similarities:** a** BLM-NII and Enzyme dataset,** b**﻿ NetLapRLS and GPCR dataset,** c** NRLMF and Nuclear Receptors dataset,** d** ﻿WNN-GIP and Ion Channel dataset.

So far we have seen that drug–drug similarities can increase the accuracy of DTI predictions. But which method of calculating chemical structure similarity between drugs is more appropriate for the DTI predictions problem? The answer shown in Tables 2, 3, 4 and 5 is clearly SIMCOMP. But the results shown in these tables are obtained by parameters tuned for SIMCOMP [3]. Therefore, we randomly selected a dataset for each method and tuned the parameters of that method for all drug–drug similarities except random similarities. Nuclear Receptors, GPCR, Ion Channel and Enzyme datasets were considered for NRLMF, NetLapRLS, WNN-GIP and BLM-NII methods respectively. The results of these experiments are illustrated in Fig. 2. The use of SIMCOMP for NetLapRLS and WNN-GIP methods gives the best AUC in GPCR and Ion Channel datasets, respectively. The AUCs and AUPRs calculated in the rest of the experiments, i.e., 75% of them, show that TanimotoPF gave better results than the rest of the similarities. In general, it can be concluded that for these datasets and these methods, TanimotoPF and SIMCOMP are more appropriate than other similarities in the DTI prediction problem.

**Fig. 3:**
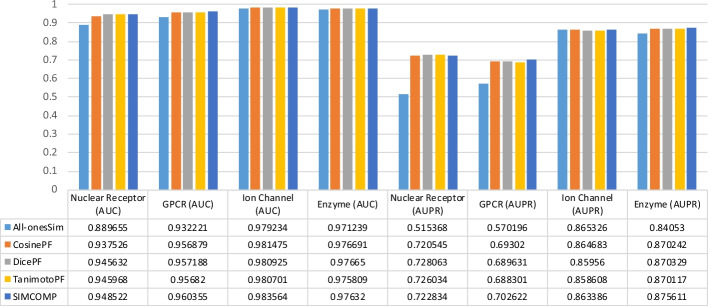
Investigating the effect of data type on the use of different drug similarities for NRLMF method

**Fig. 4:**
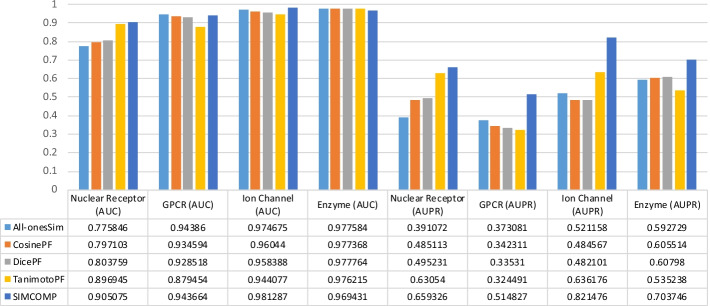
Investigating the effect of data type on the use of different drug similarities for BLM-NII method

**Fig. 5:**
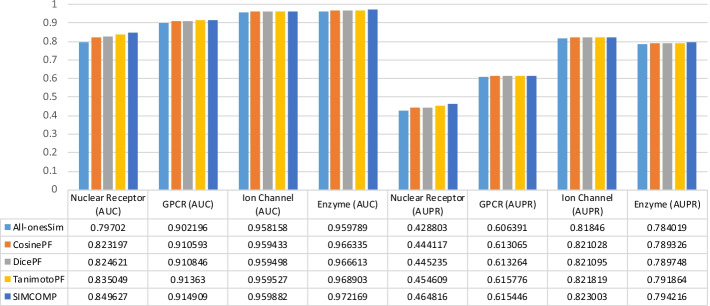
Investigating the effect of data type on the use of different drug similarities for NetLapRLS method

**Fig. 6:**
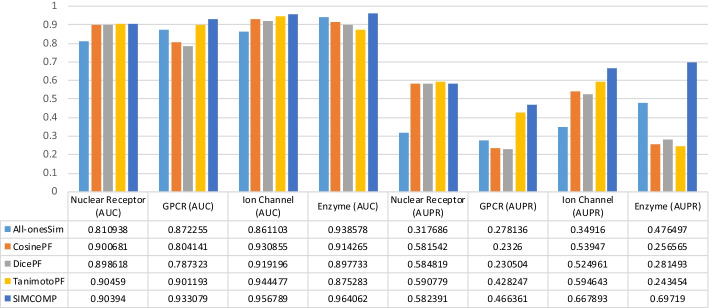
Investigating the effect of data type on the use of different drug similarities for WNN-GIP method

To investigate the effect of the type and size of the datasets on the values obtained in the experiments, we check the values in Tables 2, 3, 4 and 5 in a different way. Figures 3, 4, 5 and 6 are given for this purpose. In each figure, we considered a method and illustrated the values of AUC and AUPR obtained for that method across all datasets. The results for the NRLMF, BLM-NII, NetLapRLS and WNN-GIP methods are shown in Figs. 3, 4, 5 and 6, respectively.

The results of Figs. 3, 4, 5 and 6 can be summarized as follows:

- By replacing the similarities, the change in the value of AUPR is greater than that of AUC.
- Ion Channel and Enzyme datasets seem to be less dependent on similarity matrices replacement.
- In almost all figures, when the similarity matrix is replaced, the amount of AUC and AUPR changes for the Nuclear Receptors dataset is greater than what happens for other datasets. This has sometimes happened with less tolerance for the GPCR dataset.
- Compared to other methods, the NRLMF and NetLapRLS methods are less dependent on similarities and by replacing the matrices, their AUC and AUPR values change slightly.

In addition to the more changes that occur in the results on Nuclear Receptors and GPCR datasets, all methods perform worse on these two data, compared to other data. If we review Table 1 again, we find that these two datasets are smaller than the Ion Channel and Enzyme datasets, and the difference between the $AD_{T}$ and $AT_{D}$ criteria in these two data is a larger number. Also, the $D_{1T}$ criterion has a larger value for these two data, especially for the Nuclear Receptors dataset. Probably, these factors have caused that the different methods cannot have better performance and less tolerance on these two datasets.

We did not settle for these results and did more analysis to make sure that the impact of adding chemical structure similarities between drugs is completely related to the type and size of the data. For this purpose, for each dataset and each method, we compared the value obtained by the All-onesSim similarity matrix with its best value from Tables 2, 3, 4 and 5 and calculated the percentage of improvement. Tables 6 and 7 show these values for AUC and AUPR, respectively. What can be deduced from these tables is that, in general, the value of AUPR has improved more than that of AUC. Our datasets are all imbalances (Table 1), so it is appropriate to use the AUPR criterion for evaluation [29]. Since AUPR focuses mainly on the positive interactions, Tables 6 and 7 show that adding similarities between drugs has made the methods work better in predicting positive interactions. This improvement is quite evident in methods BLM-NII and especially WNN-GIP. The results of method WNN-GIP have improved by 46% in the lowest case and 91% in the highest case. The nature of the NRLMF and NetLapRLS methods is apparently such that the adding drug–drug similarities does not have much effect on them. As mentioned before, NRLMF works great compared to other methods. So, if its developers can make changes to the algorithm to get more impact from drug–drug similarities, then the results will be even better.

**Table 6:** Percentage of AUC improvement after considering drug–drug similarity

	Percentage improvement (enzyme) (%)	Percentage improvement (ion channels) (%)	Percentage improvement (GPCR) (%)	Percentage improvement (nuclear receptors) (%)
NRLMF	0.56	0.44	3.02	6.62
BLM-NII	0.02	0.68	0	16.66
NetLapRLS	1.29	0.18	1.41	6.6
WNN-GIP	2.72	11.11	6.97	11.55

**Table 7:** Percentage of AUPR improvement after considering drug–drug similarity

	Percentage improvement (enzyme) (%)	Percentage improvement (ion channels) (%)	Percentage improvement (GPCR) (%)	Percentage improvement (nuclear receptors) (%)
NRLMF	4.17	0	23.22	41.27
BLM-NII	18.73	57.63	37.99	68.59
NetLapRLS	1.3	0.56	1.55	8.4
WNN-GIP	46.32	91.29	67.67	85.96

**Fig. 7:**
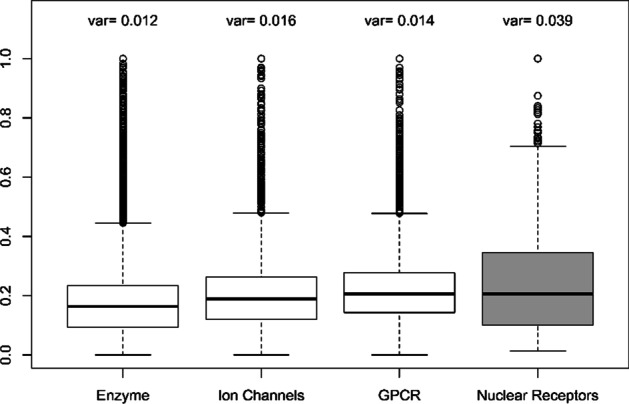
Variance and Boxplot of chemical structure similarities between drugs obtained by SIMCONP for all datasets

Another important case that can be deduced from Tables 6 and 7 is that the improvement of both the AUC and AUPR criteria for all methods in the case of Nuclear Receptors dataset is large compared to the other datasets. The size of this dataset may have caused this to happen because it is smaller than other datasets, but certainly not the only possible reason. Hence, we performed an analysis on the drug–drug similarity matrices of drugs for all datasets. Since the SIMCOMP similarities performed better in almost all Tables 2, 3, 4 and 5, we calculated the variance and drew boxplots only on these similarities. The results of this analysis are shown in Fig. 7. In this figure, the variance is denoted by $var$. It is clear that the dispersion of drug–drug similarities in Nuclear Receptors dataset, both variance and interquartile range, is greater than in other datasets. In other words, there is more information in the drug–drug similarity matrix for this dataset. Therefore, it can have a greater impact on the performance of methods, even in the case of the NRLMF and NetLapRLS methods. In fact, if the dispersion of similarities within the drug–drug matrix is low, it means that the chemical structures of the drugs are very similar, and this is equivalent to the fact that the similarities between the drugs are not considered.

## Conclusions

This paper presents a meta-analysis of adding drug–drug chemical structure similarities to DTI prediction problem. Four state-of-the-art methods were selected and implemented on four benchmark datasets. The results show that using a meaningful drug–drug similarity can improve the performance of all methods. Tables 2, 3, 4 and 5 indicated that chemical structure similarity between drugs obtained by SIMCOMP has acceptable results for almost all computational methods and all datasets. It is worth noting that these methods have some parameters which can be optimized for the different similarities.

The other important conclusion is that the improvement that occurs by adding drug–drug similarities is not the same for every dataset and every method. It strongly depends on the nature of the DTI predictor method, data type and data size. The results of a method may be greatly improved, but this improvement for another method may be negligible. Perhaps, the nature of these methods is such that the effect of adding drug–drug similarities in the processes of the various stages of their algorithm is lost and wasted. For example, the WNN-GIP method is strongly influenced by the addition of drug–drug similarities, and the results are sometimes even improved by up to 90%. But for method NRLMF, which works better than all other methods, considering drug–drug similarities has little effect on the accuracy of its predictions.

Finally, we analyzed the relationship between the datasets and the improvement discussed. We showed that if the dispersion of similarities between drugs is low then adding drug–drug similarities to the DTI problem will have little effect on improving the results. That is why the improvement of both the AUC and AUPR criteria for all methods in the case of Nuclear Receptors dataset is large compared to other datasets.

Briefly, we should mention that using drug–drug chemical structure similarity can improve the prediction results in the DTI problem. However, this improvement depends on the nature of computational predictor method, the size and type of dataset, and the type of the method used to obtain the similarities between drugs. This means that this improvement may be very small for one method and very desirable for another. It may work well on some datasets and not so much on another. If a method wants to improve the results by using the drug–drug similarities, it must increase the effect of the drug–drug similarities in some steps of its algorithm. Otherwise, it may not achieve the desired results. One direction for future work is that all the experiments performed here can be done on the target-target similarities.

## Data Availability

The datasets and codes can be freely accessed via https://github.com/Reza-HZ/DSComparisons.
